# Muographic monitoring of hydrogeomorphic changes induced by post-eruptive lahars and erosion of Sakurajima volcano

**DOI:** 10.1038/s41598-021-96947-8

**Published:** 2021-09-06

**Authors:** László Oláh, Hiroyuki K. M. Tanaka, Gergő Hamar

**Affiliations:** 1 Earthquake Research Institute, The University of Tokyo, 1-1-1 Yayoi, Bunkyo, Tokyo 113-0032 Japan; 2 International Virtual Muography Institute, Tokyo, Japan; 3 Wigner Research Centre for Physics, Eötvös Loránd Research Network, 29-33 Konkoly-Thege Miklós Str., Budapest, 1121 Hungary

**Keywords:** Natural hazards, Volcanology

## Abstract

Post-eruptive destabilization of volcanic edifices by gravity driven debris flows or erosion can catastrophically impact the landscapes, economies and human societies surrounding active volcanoes. In this work, we propose cosmic-ray muon imaging (muography) as a tool for the remote monitoring of hydrogeomorphic responses to volcano landscape disturbances. We conducted the muographic monitoring of Sakurajima volcano, Kyushu, Japan and measured continuous post-eruptive activity with over 30 lahars per year. The sensitive surface area of the Multi-Wire-Proportional-Chamber-based Muography Observation System was upgraded to 7.67 m$^2$; this made it possible for the density of tephra within the crater region to be measured in 40 days. We observed the muon flux decrease from 10 to 40% through the different regions of the crater from September 2019 to October 2020 due to the continuous deposition of tephra fallouts. In spite of the long-term mass increase, significant mass decreases were also observed after the onsets of rain-triggered lahars that induced the erosion of sedimented tephra. The first muographic observation of these post-eruptive phenomena demonstrate that this passive imaging technique has the potential to contribute to the assessment of indirect volcanic hazards.

## Introduction

Volcanic debris ejected by explosive eruptions of stratovolcanoes reach the surrounding lands by different processes^1^: (i) pyroclastic density currents are extremely hot and rapidly-moving flows of solidified lava fragments, ashes, and gases; (ii) tephra falls consist of fine (less than 2 mm in diameter) rock and lava fragments, as well as acidic aerosol doplets; (iii) lahars are fast-moving gravity-driven flows of mixture of volcanic rocks and water occurred either during eruptions or when the volcano is dormant.

The deposition of volcanic ejecta can disturb the drainage basins on volcano slopes, valleys and streams and pose indirect hazards to the surrounding landscape, economy and population within a distance approximately 30 km^1^. Preservation, erosion and secondary transport of the sedimented ejecta depend on the bulk density and the thickness of deposits, the steepness of the land, the geomorphic environment and the vegetation^2^. Wind and water driven erosion processes can destabilize and mobilize the deposited materials before they become fully incorporated into the soil. For example, the wind induced erosion of unstable newly deposited tephra from Eyjafjallajökull volcano created dust storms that seriously affected the air quality within a distance of a few hundreds of kilometers during the first summer after eruption 2010^3^. Post-eruptive lahars were generated even several years after the disturbance of drainage basins^4^ due to heavy (typically a few tens of mm per hour) rainfalls^5–7^, flooding of volcanic lakes, post-eruptive erosion of volcanic edifices, gravitational collapse of weakened structures and earthquakes^8,9^.

The generation and dynamics of lahars are controlled by the local topography, the volcanic activity, the amount and composition of tephra as well as the intensity and duration of rainfall^5,10–12^. The flows of multi-tons of material mixtures with typical volume of 10$^4$–10$^8$ m$^3$ and speed of a few tens of meters per second can even reach a distance of a few tens of kilometers from the volcanic edifice^13^. Compared with pyroclastic flows that occur within a few minutes, the lahars travel for a relatively long time: from a few tens of minutes to a few hours^14^. The speed of lahars and volume of transferred materials decrease as the distance traveled increases. The transferred materials transform to an almost hardened state after the stoppage of the flow.

Lahars can catastrophically impact the environment, such as river valleys or flood plains, human-made infrastructures, and human populations by making the lands around volcanoes uninhabitable^11,15–17^. Continuous monitoring of the amount of sedimented volcanic ejecta would allow researchers to observe the occurrence of erosion processes before the destabilization of the volcanic edifice and onset of post-eruptive lahars. Although various remote sensing techniques, such as airborne synthetic aperture radar^18^ or airborne laser scanning^19^, are utilized for measurement of long-term changes (from few months to few years) in the thickness of volcanic ejecta deposits, the monitoring of short-term (from few hours to few weeks) erosion processes has not yet been realized due to the constraints on observational time and cost.

Muography is a technique based on the measurement of the flux of penetrating cosmic-ray muons through geological edifices and human-made structures^20^ that, by a principle similar to X-ray radiography, can allow to estimate the amount of material and its evolution in time. Cosmic-ray muons are naturally occurring elementary particles that are created in the atmosphere at the altitudes of 10–15 km above sea level as decay products of hadronic particle showers. This process originates from the collisions of primary cosmic rays with atmospheric nuclei. These elementary particles can penetrate through geological structures with the thicknesses of beyond a kilometer with only a few milliradians of deflections along their paths. The applicability of muography has already been demonstrated for volcano imaging thanks to the recent progress in the development of observation instruments^21–30^ and imaging techniques^31–34^. Various volcanic phenomena, such as magma ascent and descent^35^, the degassing process^36^, plug formation^37^, magma intrusion into lava domes^38^, tectonic evolution^39^, hydrothermal processes^40,41^ or long-term tephra deposition^42^, have already been measured with muographic imaging.

In the latter muography campaign, Tanaka^42^ measured the amount of tephra on the Sakurajima volcano, Kyushu, Japan between December 2014 and November 2016 with 3 time-intervals, each lasting 240 days. The transmission rate of muons decreased more than 2 standard deviations between the 1st and 3rd intervals due to the deposition of tephra fallouts with a mass of approx 0.4 Mt. Although this muographic measurement of tephra thickness demonstrated that muography could provide useful input for the determination of the magnitude and intensity of eruptions, the limited time resolution of the experiment was not sufficient to reveal the hydrogeomorphic changes which occurred on the volcanic edifice. In this work, we exploit the technological advantages, specifically the upgraded detection acceptance and angular resolution, of the Multi-wire-proportional-chamber (MWPC)-based Muography Observation System (MMOS)^33,37,43^ of Sakurajima Muography Observatory (SMO)^33,34,37,42^ to observe the short-term erosions of the volcanic edifice that can weaken and destabilize the upper parts of the volcano. Muographic monitoring of hydrogeomorphic responses to the disturbances of volcano landscapes may contribute to the assessment of the threats posed by indirect volcanic hazards.

## Results

### Data collection

**Figure 1:**
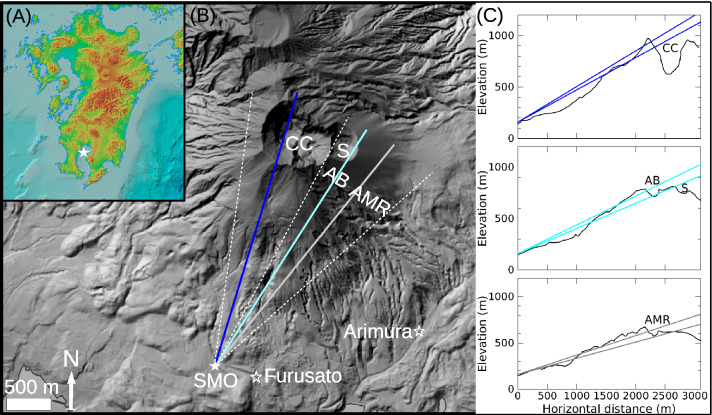
The map of the measurement site and the schematic drawing of the experimental arrangement. (**A**) The map of Kyushu, Japan is shown with the location of Sakurajima volcano (white star)^44^. (**B**) The elevation map of Sakurajima volcano is shown based on the data of Geospatial Information Authority of Japan^44^. The location of Sakurajima Muography Observatory (SMO) is shown by a filled white star. The dashed white lines show the orientation and angle of view of SMO. The coloured solid lines show selected cross-sections across the Central Craters (CC), Showa Crater (S) and Arimura Basis (AB), and Arimura Middle Reaches (AMR), respectively. The locations of lahar observation stations are shown by empty white stars. (**C**) The selected cross-sections of Sakurajima volcano are shown across the blue-coloured, cyan-coloured and gray-coloured lines of (**B**). The coloured lines indicate three selected angular regions in the vertical direction for muographic observation of the volcanic edifice.

Figure 1A–C show the location and topography of the selected experimental site at Sakurajima volcano^44^. The Sakurajima volcano is one of the world’s most active stratovolcanoes connected to magma from the Aira caldera located underneath the Kagoshima Bay^45^. Multiple craters, namely Central Craters (Vent A and B) and Showa Crater, show alternate eruptive activity with a few hundred explosive eruptions per year^46,47^. Volcano vegetation has already been damaged on the volcanic edifice due to deposition and transportation of volcanic ejecta.

Central Crater showed persistent activity since 2018 and a series of explosive eruptions have produced tephra deposition in the crater and downstream of the volcanic edifice with a typical mass of a few Mt per year^48–50^. Heavy rainfalls mobilized this tephra deposition and induced several lahar events in recent years^48–50^. Rain, strain and ultrasonic gauges, wire and pressure sensors, and load cells from the Japan Meteorological Agency (JMA) are measuring the debris flow rate and velocity, as well as the accumulated debris volume present in downstream areas of Sakurajima volcano^51,52^. We used the wire sensor data that were collected from the Arimura and Furusato rivers by in-situ lahar monitoring sensors^50^. The locations of these lahar sensors are shown by empty white stars in Fig. 1B. Although lahar sensors are applicable to characterize these events, muography has potential to complement the conventional lahar sensors by measuring the amount (mass and thickness) of tephra deposition and topographical changes that controlling the onset of post-eruptive lahars. The occurrence of the aforementioned volcanic phenomena was the reason that Sakurajima volcano was an appropriate target for studying the hydrogeomorphic responses to volcano landscape disturbances.

**Figure 2:**
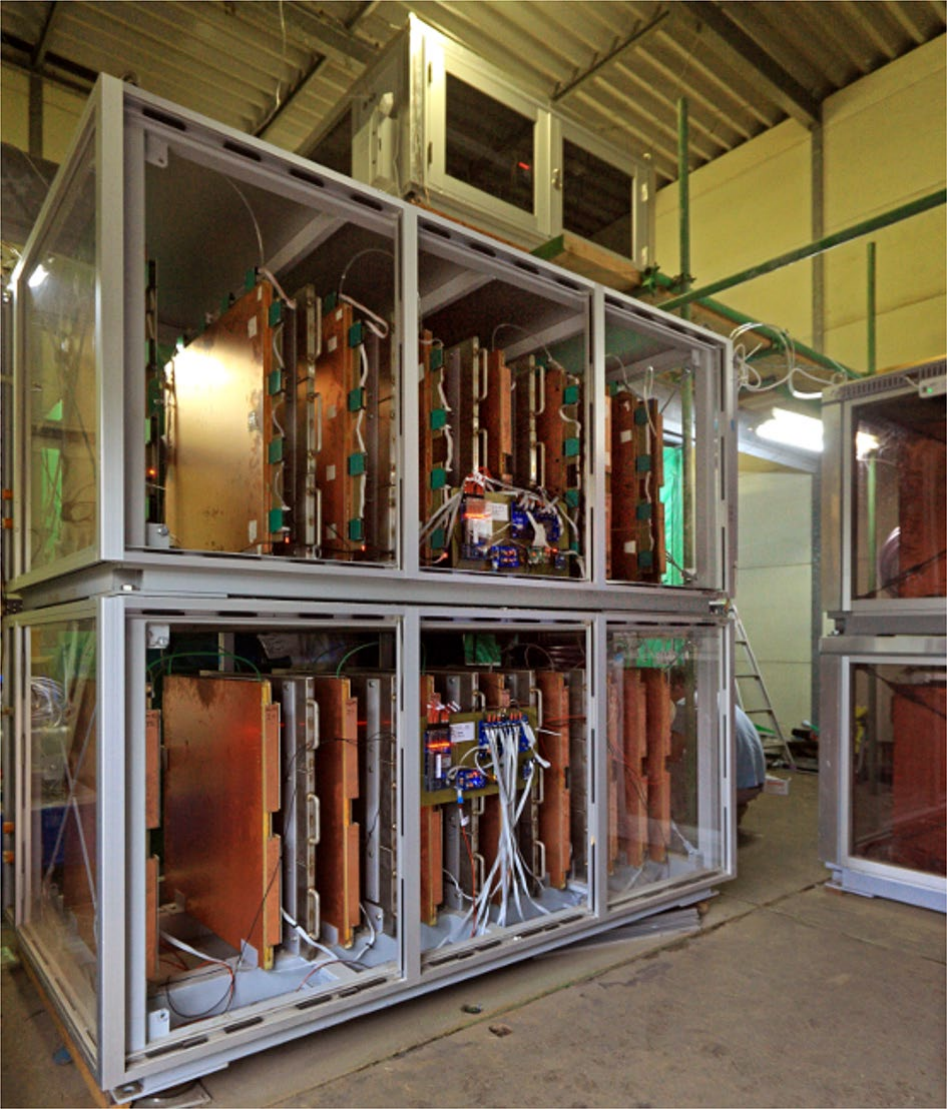
Photograph about three tracking systems of Multi-Wire-Proportional-Chamber-based Muography Observation System (MMOS). Ten MMOS operated in the Sakurajima Muography Observatory during the data collection period.

We applied the MMOS for muographic observation of Sakurajima volcano between 15th March 2019 and 18th December 2020. The MMOS was installed in the SMO that is located at 31.557$^\circ$ N, 130.650$^\circ$ E at 150 m above sea level at a distance of approx. 2800 m from the to southern peak of the volcano (indicated with the filled white stars in Fig. 1A,B). The MMOS is an autonomously operating modular muographic observation system which is serving real-time (a few-hour-time-scaled) data. A photograph of three MMOS muon tracking systems is shown in Fig. 2. The MMOS system collected data with seven tracking systems (total area of 5.31 m$^2$) for 157 days between March and August 2019 and with ten tracking systems (total sensitive surface area of 7.67 m$^2$) for 462 days between August 2019 and December 2020. The data collection procedure was stopped from the 19th to the 28th of August 2019 due to installation of the three new tracking systems. Further technical stops were performed three times with a maximum of 2 days for maintenance work. All the tracking systems were housed inside the SMO building and oriented towards the active craters of Sakurajima volcano, specifically 30.25$^{\circ }$ from the North, during the measurement campaign, as shown by dashed white lines in Fig. 1B.

### Data analysis

Since the analysis methods will be detailed in the “Methods” section of this paper, in this section the principles will be described in a nutshell. Muography exploits the relationship between the energy dependent attenuation of muons and the amount of materials along muons’ paths^53^. A projection image, called a muogram, is derived by means of angular dependent counting of the measured muon tracks. The angular dependent flux reflects the yield of penetrated muons that is quantified by normalizing of the muogram with the effective detection acceptance and data collection time. The average density-length, i.e. density integrated along the muons’ paths in the volcanic edifice, and the mass reflect the amount of materials that the muons have traveled through. These are derived by modeling or simulating of the initial angular, energy and location dependent fluxes of muons^54^ and their penetration rate through the imaged structure^53^. Based on measurements of muographic tephra monitoring campaigns, expectations are that the deposition of volcanic ejecta increase the mass of the surface region of the volcano and consequently the muons’ penetration rate and the flux decrease from this direction. In contrast, erosion processes decrease the mass of the volcanic edifice and therefore the muons’ penetration rate and the flux increase.

**Figure 3:**
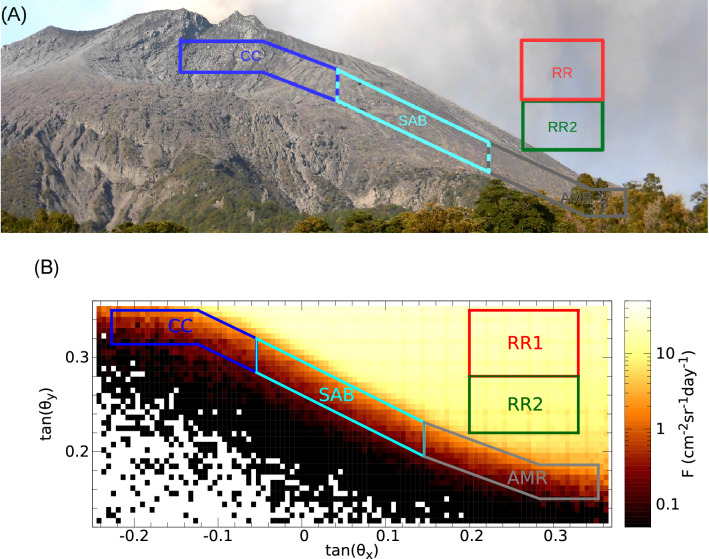
The southern peak of Sakurajima volcano is shown with the designated regions. (**A**) A photograph of the southern peak that was captured at the muography observatory. (**B**) The measured muon flux is shown as a function of horizontal (tan($\theta _x$)) and vertical (tan($\theta _y$)) directions with an angular resolution of 5.5 mrad $\times$ 5.5 mrad for a period of 227 days. The white shaded regions without flux values represent very large thicknesses with inpractically low penetrated muon count. The coloured 2D-shapes show the designated angular regions for Reference Region (RR, red), Reference Region 2 (RR2, dark-green) Central Crater (CC, blue), Showa and Arimura Basin (SAB, cyan) and Arimura Middle Reaches (AMR, gray), respectively.

In this work, an event-by-event off-line data analysis was utilized in order to reconstruct the trajectories of detected charged particles^33^. The muon flux was calculated in a tan($\theta _x$) − tan($\theta _y$) coordinate system, where tan($\theta _x$) and tan($\theta _y$) track slopes were determined relatively to the horizontal and vertical orientations of MMOS, respectively^33,37^. Four angular regions were selected for studying the muon flux changes and revealing the underlying volcanic phenomena. In Fig. 3A, coloured 2D-shapes indicate the selected regions that were selected based on the convex shape of the of volcanic edifice in a photograph of Sakurajima volcano and Fig. 3B shows a muographic image that was captured with an angular bin size of 5.5 mrad $\times$ 5.5 mrad with the MMOS system during a period of 227 days. Two reference regions (RR, red rectangle and RR2, dark-green rectangle) were designated with an open sky measurement to investigate the change of muon flux without any volcanic phenomena at different zenith-angle ranges. Three further angular regions, namely the Central Crater vents (CC, blue 2D-shape), Showa and Arimura Basin (SAB, cyan parallelogram) and Arimura Middle Reaches (AMR, gray 2D-shape), were designated through the southern peak of Sakurajima volcano. Figure1C shows the cross-sectional view of Sakurajima for different directions. The thickness of materials through the selected volcano regions were ranged from 1030 m-water-equivalent to 2630 m-water-equivalent and the land deformation due to the volcanic activity was negligible. We did not expect the muon flux to change as it transversed through the three volcano regions due to internal volcanic activity: neither due to magmatic plug formation^37^ nor explosion^34^ because we focus on the surface region. The fluxes were averaged in each angular region for time-intervals of 4 days, and the averaged fluxes were smoothed by applying a moving average calculated from the previous ten consecutive time-intervals, i.e. over a period of 40 days. In the following section, we refer to this quantity as averaged muon flux.

### Muon flux reduction due to tephra sedimentation

**Figure 4:**
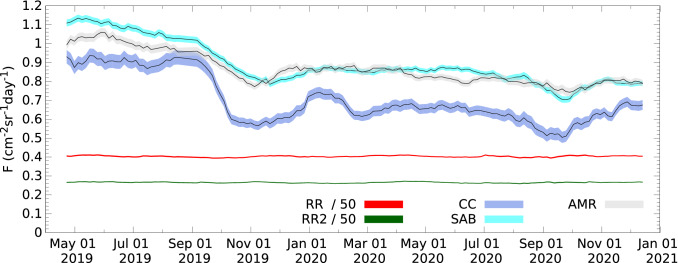
The averaged muon fluxes are shown with 1 standard deviation for the two Reference Regions (red-coloured and dark-green-coloured bands), Central Craters (blue-coloured band), Showa Crater and Arimura Basin (cyan-coloured band) and Arimura Middle Reaches (gray-coloured band), respectively.

**Figure 5:**
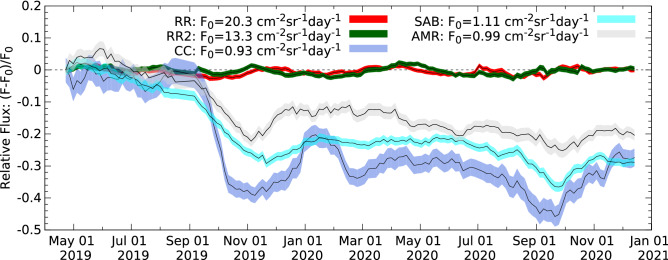
The variations of averaged muon fluxes (F) relatively to the fluxes measured during the 1st time-interval from 12th March to 21st April, 2019 (F$_0$). The relative flux changes are shown with 1 standard deviation bands during the data collection period for the two Reference Regions (red-coloured and dark-green-coloured bands), Central Craters (blue-coloured band), Showa Crater and Arimura Basin (cyan-coloured band) and Arimura Middle Reaches (gray-coloured band), respectively.

Figures 4 and 5 show respectively the averaged muon fluxes and the relative averaged muon fluxes through the five selected angular regions with 1 standard deviation bands from 21st April 2019 to 1st December 2020. It is worth noting that smaller flux was measured through the volcanic edifice at smaller zenith angles due to the higher intensity of near-horizontal muons^21^, and the detection of fewer particles that did not penetrate across the volcano^37^. The relative averaged fluxes were quantified for each region by means of the normalization of fluxes with the fluxes measured during the 1st time-interval from 12th March to 21st April, 2019 (F$_0$). The variations of relative averaged fluxes in the two reference regions (red and dark-green error bands) did not exceed the expected 1.5%  due to the atmospheric temperature and pressure changes, except when the loss of detector acceptance caused a decrease from 1.5  to 3%. To avoid the instrumental effects, the flux measured through the CC was corrected with the relative flux changes observed through RR, and the fluxes of SAB and AMR were corrected with the changes of RR2, respectively. After September 2019, the relative averaged fluxed decreased through the volcano regions from 10 to 40%. The relative flux variations were observed larger through the CC due to the direct impact of tephra deposition. The significant muon flux reduction occurred in September 2019 and then the decreasing muon flux trend reflect the change of volcanic ejecta mass that was measured to approx. 0.25 Mt between April and September 2019 and approx. 2 Mt from September 2019 to July 2020 by the Japan Meteorological Agency^48,49^.

## Discussion

**Figure 6:**
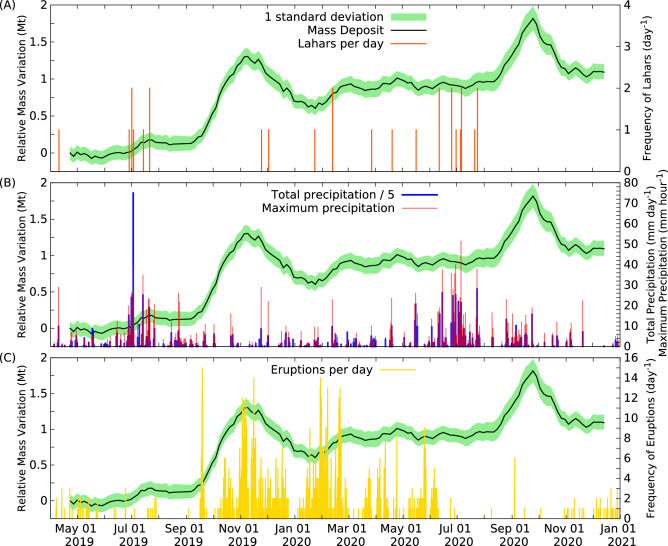
The relative variation of total mass measured through the three volcano regions is shown relative to the total mass measured during the 1st time-interval from 12th March to 21st April, 2019 as a function of time. The relative mass values (black lines) are plotted with 1 standard deviation error (green-coloured bands) and its variation is compared to the variations of the daily number of lahar events (orange-coloured impulses in **A**), total daily precipitation (blue-coloured impulses in **B**), maximum hourly precipitation (red-coloured impulses in **B**) and daily number of eruptions (yellow-coloured impulses in **C**), respectively.

Since averaged muon fluxes measured through the three volcano regions (Fig. 4) showed a similar overall trend throughout the data collection period, we quantified the total mass through these regions to present our observations on hydrogeomorphic responses to the disturbances of Sakurajima volcano. The total mass includes the mass of volcanic edifice and the mass of deposited tephra. The total mass was measured to M$_0$ = (7.54 ± 0.05) Mt during the 1st time-interval. Figure 6A–C show the variations of total mass relative to M$_0$ (M − M$_0$) with 1 standard deviation (green-coloured bands), the daily number of lahars (orange-coloured impulses), the daily total precipitation (blue-coloured impulses) and hourly maximum precipitation (red-coloured impulses), as well as the daily frequency of eruptions occurred from the Central Crater vents (yellow-coloured impulses) between 21st April 2019 and 18th December 2020. The comparison of the timelines of different quantities suggested that the onsets of lahar events were triggered by the heavy rainfalls with hourly maximum precipitation of above 10 mm per hour instead of the total daily precipitation. This is being consistent with the generation of post-eruptive lahars on Merapi^5^, Volcán de Colima^6^, and Pinatubo^7^ volcanoes. Furthermore, there was no correlation between the onset of lahars and the eruptive frequency.

We found that the mass deposition rate did not correlate with the frequency of eruptions. This observation is found to be consistent with the muographic observation of Showa cater conducted between 2014 and 2016 which suggested that the amount of ejected materials per eruption is larger when the interval between the eruptions is longer^55^. In spite of the continuous deposition of erupted tephra, the mass deposit showed significantly decreasing trends through various periods, e.g. from November 2019 to January 2020, during March 2020 and October 2020. The observed mass decreases are interpreted as follows. (i) The volcanic sediments were transported from the selected peak regions to downstream regions of the volcano by the onset of rain-triggered lahar events. (ii) Further mass decreases were observed without the occurrence of lahar events, e.g. after mid-September 2020. This observation suggests the water driven erosion of the peak region of Sakurajima volcano. These processes were induced by rain falls that were observed continuously during the typhoon season from August to October 2020. It is worth noting that the mass changes did not reflect all lahars. On the one hand, these lahars were assumed to have been initiated in the downstream region after the erosion of peak region. On the other hand, the slightly increasing mass from February 2020 to August 2020 with a series of lahar events hints that the muographic observation of erosion process is limited when the tephra deposition extinguish or overwhelm the mass decreasing processes. In spite of this limitation, muographic observation of mass changes on the volcanic edifice has potential to reveal the topographical changes that can trigger the onset of lahars which can not be observed by conventional lahar sensors. Furthermore, muography can measure the amount of deposited materials that can be remobilized by future lahars, and thus it can contribute to the mitigation of lahars.

In conclusion, we propose muography as a tool for monitoring of the hydrogeomorphic responses to the disturbance of the landscape of Sakurajima volcano. The sensitive surface area of the MMOS system was upgraded up to 7.67 m$^2$ to detect muons within a total acceptance of 1.36 m$^2$sr. This drastically decreased the time required to measure the volume of materials, either tephra deposition or transportation, through the crater regions from 240 to 40 days. The measured muon flux and mass deposit changes reflected the tephra deposition which occurred due to the ejection of approx. 2 Mt tephra after October 2019, as well as tephra transport by post-eruptive lahars and rain-triggered erosion of the volcanic edifice. These results demonstrate the applicability of muography for the measurement of erosion processes at a shorter duration, which may contribute to improved modeling of the erosion of the volcanic edifice and assessment of volcano hazard levels.

## Methods

### Observational instruments

**Figure 7:**
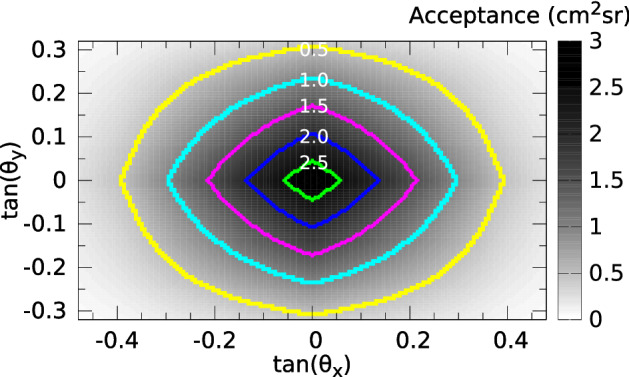
The total acceptance of ten tracking systems is shown as a function of horizontal and vertical track slopes with a bin size of approx. 5.5 mrad $\times$ 5.5 mrad. Coloured lines highlight equal values at the acceptance of 0.5 cm$^2$sr (yellow), 1 cm$^2$sr (cyan), 1.5 cm$^2$sr (pink), 2 cm$^2$sr (blue) and 2.5 cm$^2$sr (green), respectively.

 The design, operation and performance of the MWPCs and the tracking systems are presented extensively in Refs.^28,33,43^, therefore here just a brief description is provided. Each tracking system was assembled with seven or eight MWPCs comprising a total surface area of 0.64 m$^2$ or 0.96 m$^2$. The length of each tracking system was set to approx. 2 m. The total effective acceptance of ten tracking systems was 1.38 m$^2$sr. Figure 7 shows the merged acceptance of MMOS system as a function of track slopes. Five 2-cm-thick lead plates were installed between the tracking layers to deflect or absorb the low-energy particles. This setup of MWPCs and lead plates provided angular resolutions of approx. 3 mrad for both horizontal and vertical directions^33^.

Micro-computer-based detector control and data acquisition systems were installed in each tracking system to achieve autonomous operation. The data collection was triggered by a coincidence of at least three MWPCs. The triggering efficiency was found above 99%  for each tracking system and trigger was blocked for a few hundreds of microseconds after acquiring a signal during the data readout. This resulted in a dead time of approx. 0.2%  for each tracking system during the data collection period. A local server micro-computer communicated with the micro-computers of tracking systems and controlled the data collection and management. The collected data were transferred to a remote server where data storage and quality assurance were performed. The maintenance of the MMOS was required regularly every 4–6 months.

### Track reconstruction and flux measurement

 Track reconstruction and selection were performed by an event-by-event analysis independently for each MMOS module^33^. A pre-analysis was applied for exclusion of noisy readout channels and alignment of detector layers. The tracks were reconstructed by means of two independent line fits on the reconstructed cluster centroids in horizontal and vertical directions. The tracks were selected from the reconstructed set based on the chi-squares of fitted lines^33,37^. The flux of the module ($F_\text {mod}$) was calculated for each tan($\theta _x$) − tan($\theta _y$) angular bin as follows.

1 $$\begin{aligned} F_\text {mod} = \frac{ N_{\text {tracks}} }{ A \times \Delta t }\,\,\,, \end{aligned}$$

where $N_{\text {tracks}}$ is the number of selected tracks, *A* is the acceptance of tracking system, $\Delta t$ is the effective data collection time. The horizontal orientations of different tracking systems were found within an angle of 12 mrad relative to a reference one, namely MMOS01, by means of minimization of relative flux differences^37^. These corrections were also taken into account in the track reconstruction procedure. In the last step of flux calculation, the $F_\text {mod}$ were weighted with the relative flux errors and averaged to quantify the merged flux of MMOS system (F).

### Mass calculation

**Figure 8:**
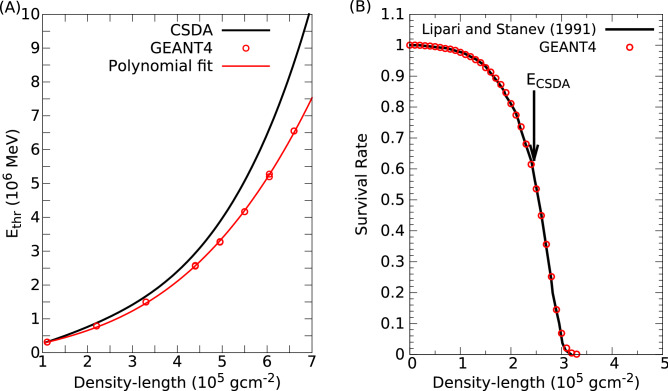
The threshold energies of muons were quantified with GEANT4 simulation. (**A**) The continuous-slowing-down-approximation^53^ (CSDA, black line) and the simulated threshold energies (red circles) are shown for silicon dioxide. Red line shows the fitted polynomial function. (**B**) The survival rates of muons with an energy of 1 TeV through standard rock were found in agreement for data produced by Monte Carlo simulations presented in Ref.^56^ (black line) and GEANT4 (red circles). Black arrow shows the range of 1 TeV muon in standard rock quantified by CSDA.

 The *X* density-lengths were determined by means of the comparison of measured and modeled fluxes. The latter ones were derived to various density-lengths up to 3000 m-water-equivalent with a step of 2 m-water-equivalent as follows.

2 $$\begin{aligned} F_{calc}(90^{\circ }-\theta _{y}, L) = \int _{\text {E}_{\text {thr}}(L)}^{\infty } f_{diff}(E, 90^{\circ }-\theta _{y}) {{\mathrm {d}} E}\,\,\,, \end{aligned}$$

where $f_{diff}(E, 90^{\circ }-\theta _{y})$ is the differential muon energy spectra that were modeled by a modified Gaisser parametrization^54^, and E$_{\text {thr}}(L)$ is the threshold energy that is required to muons to penetrate *L* density-length. The fluctuations of radiative energy loss processes dominate the energy loss of muons^56^ that are being able to penetrate the volcanic edifice. E$_{\text {thr}}$(L) was parametrized with the procedure proposed in Ref.^57^. Muons were generated with realistic muon spectra at the zenith-angles of 75$^{\circ }$ and 79$^{\circ }$, and their penetration strengths were simulated through silicon dioxide walls with different density-lengths in GEANT4 framework^58^. The extracted threshold energies were fitted in a density-length range of (1–7)$\times$10$^5$ g cm$^{-2}$ with a polynomial function: E$_{\text {thr}}(L)\left[ \text {MeV}\right] = p_1L+p_3L^3$, where the *L* is given in g cm$^{-2}$ units and $p_1$ = 2.62 ± 0.06, $p_3$ = (165.98 ± 1.88)$\times$10$^{-13}$. Figure 8A shows the E$_{\text {thr}}(L)$ in silicon dioxide calculated by continuous-slowing-down-approximation (CSDA, black line)^53^ and determined by GEANT4 (red circles). Figure 8B shows the survival rates of 1-TeV-muons through standard rock ($\rho$ = 2.65 g cm$^{-3}$, A = 22, Z = 11) for the data generated by GEANT4 (red circles) and a simulation presented in Ref.^56^ (black line). Data produced by different simulations were found in agreement; this result validated the simulation presented in this work. The *L* density-lengths were extracted when differences between the measured and modeled fluxes were found to be minimal. The average masses, *M*, through the designated volcano regions were estimated using the following formula^42^:

3 $$\begin{aligned} M = N \times D^2 \times \Delta \text {tan}\left( \theta _x\right) \times \Delta \text {tan}\left( \theta _y\right) \times L\,\,\,, \end{aligned}$$

where *N* is the number of angular bins that were involved in the designated region, $D \approx$ 2700 m is the distance between the MMOS and volcanic edifice, as well as tan$(\theta _x) \ll 1$ and tan$(\theta _y) \ll 1$.

### Atmospheric effects of muon production

**Figure 9:**
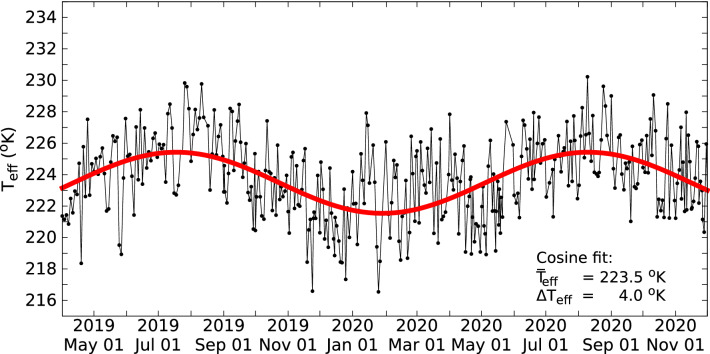
The effective temperatures are shown for the data collection period. The T$_{\text {eff}}$ values were calculated using the balloon-borne measurements performed above the Kagoshima Bay^62^. The cosine fit shows an average seasonal variation of 4 $^{\circ }$K around the average value of 223.5 $^{\circ }$K.

 Changes in atmosphere’s thermal state affect the muon production^59,60^ that has to be taken into account in muographic monitoring^40^. The $\Delta$F change from the F muon flux due to pressure and temperature changes can be quantified with the following empirical formula:

4 $$\begin{aligned} \frac{\Delta \text {F}}{\text {F}} = \beta \Delta p + \alpha _T \frac{\Delta \text {T}_{\text {eff}}}{T_{\text {eff}}}\,\,\,, \end{aligned}$$

where $\beta$ is the barometric coefficient, $\Delta$p is the pressure change from the average pressure that is measured at the observational site, $\alpha _T$ is the temperature coefficient that depends on amount of transferred materials and T$_{\text {eff}}$ is the weighted average of temperatures measured under different atmospheric pressures. The temperature weights describe the propagation of pions and kaons through the atmosphere^61^. The pressure and effective temperature changes were quantified using the atmospheric weather data that were collected above the Kagoshima Bay^62^. The average pressure was found to be 986 hPa with a change of $\Delta p$ < 10 hPa. The $\beta$ was estimated below 10$^{-5}$ hPa$^{-1}$ for TeV muons^63^, thus the barometric effect was estimated to well below 1%. The effective temperatures were quantified with the procedure that was proposed in Ref.^61^. The energy threshold and the zenith-angle parameters were set to 500 GeV and 75$^{\circ }$. The seasonal variation of effective temperature is shown by blue dots in Fig. 9. These were fitted with a cosine function (solid red line) and an effective temperature variation of 4 $^{\circ }$K was quantified around the average effective temperature of 223.5 $^{\circ }$K. The corresponding coefficient was approximated to $\alpha _{T}~\approx$ 1 using the data compiled in Ref.^61^. Therefore, a relative muon flux variation of 1.5% was estimated for our campaign due to atmospheric pressure and temperature effects.

## Data Availability

The datasets generated during and/or analyzed during the current study are available from the corresponding author on reasonable request.

## References

[1] Pierson TC, Major JJ (2014). Hydrogeomorphic effects of explosive volcanic eruptions on drainage basins. Annu. Rev. Earth Planet. Sci..

[2] Blong R, Enright N, Grasso P (2017). Preservation of thin tephra. J. Appl. Volcanol..

[3] Arnalds O (2013). An extreme wind erosion event of the fresh Eyjafjallajökull 2010 volcanic ash. Sci. Rep..

[4] Waldron HH (1967). Debris flow and erosion control problems caused by the ash eruptions of Irazú Volcano, Costa Rica. USGS Bull..

[5] Lavigne F, Thouret JC, Voight B, Suwa H, Sumaryono A (2000). Lahars at Merapi volcano, Central Java: An overview. J. Volcanol. Geotherm. Res..

[6] Capra L (2010). Rainfall-triggered lahars at Volcán de Colima, Mexico: Surface hydro-repellency as initiation process. J. Volcanol. Geotherm. Res..

[7] van Westen JC, Daag AS (2005). Analysing the relation between rainfall characteristics and lahar activity at Mount Pinatubo, Philippines. Earth Surf. Process. Landf..

[8] Pierson TC, Wood NJ, Driedger CL (2014). Reducing risk from lahar hazards: Concepts, case studies, and roles for scientists. J. Appl. Volcanol..

[9] Vallance JW (2005). Volcanic debris flows. Hazards and Related Phenomena. Springer Praxis Books.

[10] Kurokawa AK, Ishibashi H, Miwa T, Nanayama F (2018). Rheological behavior of water-ash mixtures from Sakurajima and Ontake volcanoes: Implications for lahar flow dynamics. Bull. Volcanol..

[11] Pierson TC (1985). Initiation and flow behavior of the 1980 Pine Creek and Muddy River lahars, Mount St. Helens, Washington. Geol. Soc. Am. Bull..

[12] Courtland LM (2012). GPR investigation of tephra fallout, Cerro Negro volcano, Nicaragua: A method for constraining parameters used in tephra sedimentation models. Bull. Volcanol..

[13] Iverson RM, Schilling PS, Vallance JW (1998). Objective delineation of lahar-inundation hazard zones. Geol. Soc. Am. Bull..

[14] Zobin VM, Plascencia I, Reyes G, Navarro C (2009). The characteristics of seismic signals produced by lahars and pyroclastic flows: Volcán de Colima, México. J. Volcanol. Geotherm. Res..

[15] Lowe D (1986). Lahars initiated by the 13 November 1985 eruption of Nevado del Ruiz, Colombia. Nature.

[16] U.S. Geological Survey. The Cataclysmic Eruption of Mount Pinatubo, Philippines. *U.S. Geological Survey Fact Sheet* 113–197 https://pubs.usgs.gov/fs/1997/fs113-97/ (1991).

[17] Kerle N, van Wyk de Vries B (2001). The debris avalanche at Casita volcano, Nicaragua—Investigation of structural deformation as the cause of slope instability using remote sensing. J. Volcanol. Geotherm. Res..

[18] Bamler R, Hartl P (1998). Synthetic aperture radar interferometry. Inverse Probl..

[19] Wehr A, Lohr U (1999). Airborne laser scanning—An introduction and overview. ISPRS J. Photogramm. Remote Sens..

[20] George EP (1955). Cosmic rays measure overburden of tunnel. Commonw. Eng..

[21] Tanaka HKM (2007). High resolution imaging in the inhomogeneous crust with cosmic-ray muon radiography: The density structure below the volcanic crater floor of Mt. Asama. Japan. Earth Planet. Sci. Lett..

[22] Tioukov V (2019). First muography of Stromboli volcano. Sci. Rep..

[23] Marteau J (2017). DIAPHANE: Muon tomography applied to volcanoes, civil engineering, archaelogy. JINST.

[24] Saracino G (2017). The MURAVES muon telescope: Technology and expected performances. Ann. Geophys..

[25] Kusagaya T, Tanaka HKM (2015). Muographic imaging with a multi-layered telescope and its application to the study of the subsurface structure of a volcano. Proc. Jpn. Acad. Ser. B.

[26] Lo Presti D (2018). The MEV project: Design and testing of a new high-resolution telescope for muography of Etna Volcano. Nucl. Instrum. Methods Phys. Res. Sect. A.

[27] Cârloganu C (2013). Towards a muon radiography of the Puy de D$$\hat{\rm o\it }$$me.. Geosci. Instrum. Method Data Syst..

[28] Varga D, Nyitrai G, Hamar G, Oláh L (2016). High efficiency gaseous tracking detector for cosmic muon radiography. Adv. HEP..

[29] Catalano O (2016). Volcanoes muon imaging using cherenkov telescopes. Nucl. Instrum. Methods Phys. Res. Sect. A.

[30] Peña-Rodríguez J (2020). Design and construction of MuTe: A hybrid Muon Telescope to study Colombian volcanoes. JINST.

[31] Nishiyama R (2014). Integrated processing of muon radiography and gravity anomaly data toward the realization of high-resolution 3-D density structural analysis of volcanoes: Case study of Showa-Shinzan lava dome. Usu. Japan. JGR Solid Earth.

[32] Tanaka HKM (2016). Instant snapshot of the internal structure of Unzen lava dome, Japan with airborne muography. Sci. Rep..

[33] Oláh L, Tanaka HKM, Ohminato T, Varga D (2018). High-definition and low-noise muography of the Sakurajima volcano with gaseous tracking detectors. Sci. Rep..

[34] Nomura Y (2020). Pilot study of eruption forecasting with muography using convolutional neural network. Sci. Rep..

[35] Tanaka HKM, Kusagaya T, Shinohara H (2014). Radiographic visualization of magma dynamics in an erupting volcano. Nat. Commun..

[36] Tanaka HKM, Uchida T, Tanaka M, Shinohara H, Taira H (2009). Cosmic-ray muon imaging of magma in a conduit: Degassing process of Satsuma-Iwojima Volcano. Jpn. Geophys. Res. Lett..

[37] Oláh L, Tanaka HKM, Ohminato T, Hamar G, Varga D (2019). Plug formation imaged beneath the active craters of Sakurajima volcano with muography. Geophys. Res. Lett..

[38] Barnoud A (2021). Robust Bayesian joint inversion of gravimetric and muographic data for the density imaging of the Puy de Dôme Volcano (France). Front. Earth Sci..

[39] Lo Presti D (2020). Muographic monitoring of the volcano-tectonic evolution of Mount Etna. Sci. Rep..

[40] Jourde K, Gibert D, Marteau J, de Bremond d’Ars J, Komorowski J-C (2016). Muon dynamic radiography of density changes induced by hydrothermal activity at the La Soufriére of Guadeloupe volcano. Sci. Rep..

[41] Le Gonidec Y (2019). Abrupt changes of hydrothermal activity in a lava dome detected by combined seismic and muon monitoring. Sci. Rep..

[42] Tanaka HKM (2020). Development of the muographic tephra deposit monitoring system. Sci. Rep..

[43] Varga D (2020). Detector developments for high performance muography applications. Nucl. Instrum. Methods Phys. Res. Sect. A.

[44] Geospatial Information Authority of Japan. http://www.gsi.go.jp/ (2021).

[45] Aramaki S (1984). Formation of the Aira Caldera, southern Kyushu, $$\sim $$22,000 years ago. J. Geophys. Res. Solid Earth.

[46] Iguchi M, Yakiwara H, Tameguri T, Hendrasto M, Hirabayashi J (2008). Mechanism of explosive eruption revealed by geophysical observations at the Sakurajima, Suwanosejima and Semeru volcanoes. J. Volcanol. Geotherm. Res..

[47] Kazahaya R, Shinohara H, Mori T, Iguchi M, Yokoo A (2016). Pre-eruptive inflation caused by gas accumulation: Insight from detailed gas flux variation at Sakurajima volcano. Jpn. Geophys. Res. Lett..

[48] Japan Meteorological Agency, Report of Coordinating Committee for Prediction of Volcanic Eruption No. 145. https://www.data.jma.go.jp/svd/vois/data/tokyo/STOCK/kaisetsu/CCPVE/shiryo/145/145_2.pdf (2019).

[49] Japan Meteorological Agency, Report of Coordinating Committee for Prediction of Volcanic Eruption No. 146. https://www.data.jma.go.jp/svd/vois/data/tokyo/STOCK/kaisetsu/CCPVE/shiryo/146/146_2-1.pdf (2020).

[50] Japan Meteorological Agency, Report of Coordinating Committee for Prediction of Volcanic Eruption No. 147. https://www.data.jma.go.jp/svd/vois/data/tokyo/STOCK/kaisetsu/CCPVE/shiryo/147/147_2-1.pdf (2020).

[51] Takeshi T (2011). Evolution of Debris-flow Monitoring Methods on Sakurajima. Int. J. Erosion Control Eng..

[52] Osaka, T., Utsonomiya, R., Tagata, S., Itoh, T., & Mizuyama, T. Debris Flow Monitoring using load cells and pressure sensors in Sakura-jima Island *7th International Conference on Debris-Flow Hazards Mitigation Proceedings*10.25676/11124/173226 (2019).

[53] Groom DE, Mokhov NV, Striganov SI (2002). Muon stopping power and range tables 10 MeV–100 TeV. Atomic Data Nucl. Data Tables.

[54] Tang A, Horton-Smith G, Kudryavtsev VA, Tonazzo A (2006). Muon simulations for Super-Kamiokande, KamLAND, and CHOOZ. Phys. Rev. D.

[55] Kusagaya, T. *Reduction of background noise in muographic images for detecting magma dynamics in an active volcano*. Phd Thesis, The University of Tokyo 10.15083/00077464 (2017).

[56] Lipari P, Stanev T (1991). Propagation of multi-TeV muons. Phys. Rev. D.

[57] Oláh, L. & Tanaka, H. K. M. Muography of magma intrusion beneath the active craters of Sakurajima volcano. In *Muography: Exploring Earth’s Subsurface with Elementary Particles* (eds Oláh, L. *et al.*) Ch. 8 (AGU Geophysical Monograph Series 270, 2021).

[58] Agostinelli S (2003). Geant4—A simulation toolkit. Nucl. Instrum. Meth. A.

[59] Cecchini S, Spurio M (2012). Atmospheric muons: experimental aspects. Geosci. Instrum. Method. Data Syst..

[60] Tramontini M, Rosas-Carbajal M, Nussbaum C, Gibert D, Marteau J (2019). Middle-atmosphere dynamics observed with a portable muon detector. Earth Space Sci..

[61] Adamson P (2010). Observation of muon intensity variations by season with the MINOS far detector. Phys. Rev. D.

[62] University of Wyoming. http://weather.uwyo.edu/upperair/sounding.html (2021).

[63] Sagisaka S (1986). Atmospheric effects on cosmic-ray muon intensities at deep underground depths. Il Nuovo Cimento.

